# Study of ongoing registered clinical trials on COVID-19: a narrative review

**DOI:** 10.1590/1516-3180.2020.0208.R1.15062020

**Published:** 2020-08-14

**Authors:** Md Insiat Islam Rabby, Farzad Hossain

**Affiliations:** I BSc, Engineer and Master’s Student, Department of Mechanical and Manufacturing Engineering, Universiti Putra Malaysia, Seri Kembangan, Selangor, Malaysia.; II BSc, Engineer and Master’s Student, Department of Mechanical and Production Engineering, Islamic University of Technology, Gazipur, Bangladesh.

**Keywords:** SARS virus, Therapeutics, Vaccines, Records, Novel coronavirus, Diagnostic test, Devices, Biological

## Abstract

**BACKGROUND:**

The dangerous SARS-CoV-2 virus first emerged in China in December 2019 and has rapidly spread worldwide. Currently, it has affected more than 2,850,000 people. No vaccine or drug is available yet, and therefore researchers and scientists are striving to identify potential drugs or vaccines for combating this virus. We were unable to find any review of the literature or analysis on ongoing registered clinical trials that reported diagnostic tests, therapeutics, vaccines and devices for COVID-19 along with estimated enrollment, participants’ ages, study type, start and completion date, status, treatment/intervention and country.

**OBJECTIVE:**

To review ongoing trials relating to COVID-19.

**METHODS:**

A systematic search for clinical trials was conducted in the ClinicalTrials.gov database up to April 12, 2020. A total of 339 trials relating to COVID-19 were analyzed and key information on each trial was recorded.

**RESULTS:**

Most of the trials were being conducted in the United States and completion of most of them was expected by May 2020. They were mostly on drugs and treatment, while a minority were on diagnostic tests. The analysis showed that hydroxychloroquine was investigated in most of the trials. The trials identified were categorized into five classes: a) diagnostic tests; b) therapeutics; c) biologics and vaccines; d) devices and products; and e) others.

**CONCLUSION:**

The trials identified have potential against COVID-19 that can be applied in treatment processes after the necessary investigations and experiments. Additionally, the items identified were organized in a proper way, which can assist in current research activities.

## INTRODUCTION

The novel coronavirus (SARS-CoV-2) originated from Wuhan, in Hubei Province, China, and it has spread across more than 28 countries with more than 25,000 confirmed cases and around 500 deaths from mid-December 2019 to early February 2020.^1^ Within that period, the case-fatality rate was around 2% and over 90% of the deaths and cases were in China.^1^ Moreover, the majority of them were males with an average age of 55 years, according to reports on the initial surge of cases in Wuhan, which were linked to the Huanan Seafood Wholesale Market.^2^ Almost similar symptoms (i.e. coughing, fever, myalgia and fatigue) were reported in most of the cases.^3^ Pneumonia and some other serious and even fatal respiratory diseases (i.e. acute respiratory distress syndrome) were developed in the majority of the cases.^3^

The 2019 novel coronavirus (SARS-CoV-2) is a beta coronavirus and it forms a clade within the subgenus Sarbecovirus of the subfamily Orthocoronavirinae.^4^ Outbreaks of some other beta coronaviruses of zoonotic origin, i.e. Middle East respiratory syndrome coronavirus (MERS-CoV) and severe acute respiratory syndrome coronavirus (SARS-CoV) occurred previously, in 2012 and 2003 respectively, and were linked to potentially fatal illness.^5^^,^^6^ Around 3% pathogenicity has been observed in relation to SARS-CoV-2 according to the current evidence and this is comparatively lower than the rates for MERS-CoV (40%) and SARS-CoV (10%).^7^ However, potentially higher transmissibility (R0: 1.4-5.5) has been observed for SARS-CoV-2, whereas it was only (R0: < 1) and (R0: 2-5) for MERS-CoV and SARS-CoV respectively.^7^

SARS-CoV-2 has the possibility of expansion globally and the World Health Organization has already declared it to be a Public Health Emergency of International Concern.^8^ In this situation, rapid diagnostics, drugs and vaccines have become urgent necessities for promptly detecting, preventing and containing SARS-CoV-2. Potential quick diagnostics, drugs and vaccines for SARS-CoV-2 have been described and assessed in systematic reviews. A few studies on clinical trials relating to COVID-19 (the disease that the novel coronavirus causes) are already in the literature, but these are not enough, given the current situation. These trials only focused on drugs and were also limited to specific regions.^9^^,^^10^^,^^11^^,^^12^ No clinical trials on diagnostic tests, devices, vaccines, biologics, behavior and other matters have yet been reported in the literature. The present study identified and discussed all potential categories of registered clinical trials on COVID-19 in ClinicalTrials.gov database up to April 12, 2020. Additionally, statistical analysis based on the findings was also conducted.

## OBJECTIVE

To create a complete study focusing on all categories of clinical trials relating to COVID-19, which is a necessity for assisting the current COVID-19 research activities.

## METHODS

The necessary data were collected by searching ClinicalTrials.gov database up to April 12, 2020, using the descriptor [coronavirus] in the simple search field “conditions or disease”, without restrictions on languages, disease conditions, results or locations. The details of the search strategy are shown in Table 1. Our search also included trials that were shown with the status “recruiting” and “not yet recruiting”. On the other hand, trials for which the status was shown as “enrolling by invitation”, “active, not recruiting”, “suspended”, “terminated”, “completed”, “withdrawn” or “unknown” were not included in this study.

**Table 1.** Search strategy

#1 MeSH descriptor: [coronavirus] explode all trees = 280
#2 (COVID-19) OR (SARS-CoV) = 607
#3 #1 OR #2 = 887
Filters: in Trials Review; in *Title, Status, Interventions, Conditions* = 339

Thus, every trial was defined in terms of its specific identification number, estimated enrollment, participants’ ages, study type, start and completion date, status, treatment/intervention and country. From the information available in the database, we recorded and compared the continents of the clinical trials, total numbers of trials in various countries, expected completion time of the trials, phase of the trials, trial status, study type of the trials, estimated enrollment of participants in the trials, participants’ ages and types of intervention or treatment used in the trial. We also analyzed the registered diagnostic tests, drugs, biologics and vaccines, devices and products, and behavioral and other clinical trials relating to COVID-19.

## RESULTS

Currently, there are no specific remedies or vaccines for COVID-19 infection. Therefore, over the past few months, a huge number of clinical trials have been registered in the ClinicalTrials.gov database with the aim of identifying the most effective treatment and vaccine for COVID-19. This number is increasing continuously.

**Figure 1.**
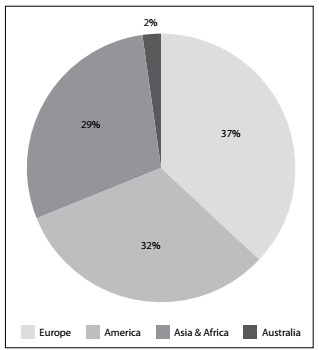
Continents of the clinical trials on COVID-19.

Our search in ClinicalTrials.gov identified 339 clinical trials on COVID-19. Figure 1 shows the continents on which these trials were conducted. From this, it was observed that the largest proportion of the clinical trials (37%) were registered in Europe, while a minority (2%) were registered in Australia.

**Figure 2.**
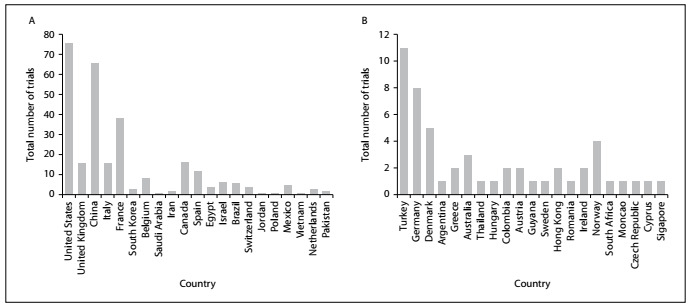
A. Total number of trials in various countries; B. Total number of trials in various countries (Continuation).

Figures 2A and B exhibit the range of the total numbers of trials among different countries. From these figures, it can be seen that the highest number of clinical trials (76) was registered in the United States and the second highest number (66) was registered in China. Meanwhile, only one trial was registered from Pakistan, Saudi Arabia, Jordan, Poland, Vietnam, Singapore, Romania, Guyana, Thailand, South Africa, Monaco, Argentina, Czech Republic, Hungary and Cyprus.

**Figure 3.**
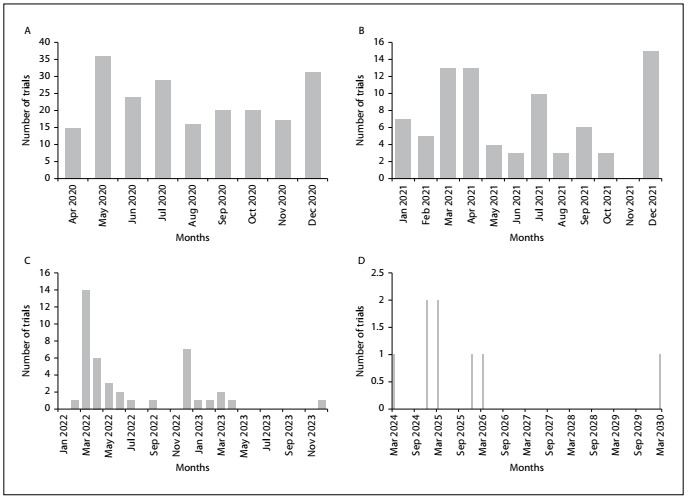
A. Expected completion dates of trials in 2020; B. Expected completion dates of trials in 2021; C. Expected completion dates of trials in 2022 and 2023; D. Expected completion dates of trials from 2024 to 2030.

Figures 3A, B, C and D show the expected completion dates of the trials. The data show that the completion dates for these trials ranged from April 2020 to approximately the year 2030. It was observed that the largest proportion of these trials (36) were expected to be completed by May 2020. However, most of these trials were expected to finish by December 2021 and more than 200 trials were expected to finish by December 2020. Some trials were expected to finish in 2025 or 2026, and there was one trial that was supposed to be completed by March 2030, relating to “observation of behavior and COVID-19 infection”. Therefore, the world still needs to wait for a certain time period, for effective results to be reached from these registered trials.

It was observed regarding the trial phase that the largest proportion of them (33%) related to phase 2, while a minority (4%) related to early phase 1. It was found from the trial status that most of them (56%) were not yet recruiting, while a minority (44%) were already recruiting. It was observed regarding the study type that most of the trials (73%) were interventional, while a minority (27%) were observational. It was found from the estimated enrollment of participants in the trial that most of the trials (46%) were planned to have enrollment of less than 500, but greater than or equal to 100. On the other hand, a minority (11%) were planned to have enrollment greater than or equal to 1000. It was observed from the participants’ ages that most of the participants (85%) were within the ‘18 years and older’ category whereas a minority (1%) were within the ‘up to 18 years old’ category. Using categories of intervention or treatment, the trials could be categorized into five classes: a) diagnostic tests; b) therapeutics; c) biologics and vaccines; d) devices and products; and e) others. In addition, it was observed from the categories of intervention or treatment that most of the trials (56%) were related to therapeutics (drugs and treatment), while a minority of the trials (7%) were related to diagnostic tests.

### Diagnostic tests

According to the United States Centers for Disease Control and Prevention, specimens should be collected by healthcare professionals not only from the lower respiratory tract (through either bronchoalveolar lavage or an endotracheal tube) but also from the upper respiratory tract (either oropharyngeal or nasopharyngeal). The diagnosis of COVID-19 pneumonia is mainly dependent on RT-PCR investigation on specimens. Serological tests can be considered if RT-PCR is unavailable.

A commercial qualitative testing system for SARS-CoV-2 using the cobas^®^ system (Roche, Basel, Switzerland) has now been approved by the United States Food and Drug Administration (FDA). The test needs samples from oropharyngeal or nasopharyngeal swabs, and the result can be obtained within 3.5 hours. The cobas^®^ SARS-CoV-2 test is a kind of double target assessment test depending upon the RT-PCR methodology. It can detect not only the particular SARS-CoV-2 ribonucleic acid but also the extremely conserved part of the invariant E gene in every member of the Sarbecovirus subgenus. To ensure accuracy and specificity, the assay comprises a comprehensive process with internal control, positive control and negative control.

Moreover, permission for urgent use of the Xpert Xpress SARS-CoV-2 test (Cepheid Inc, California, United States) was granted by the United States Food and Drug Administration (FDA) on March 21, 2020. This is another qualitative test, from which results can be obtained within 45 minutes. Whenever more than one targeted gene is detected, the results should be treated as positive. At present, the screening methods depend upon appearance of plenty of viral genomes at the sample collection site. Studies have revealed that high levels of immunoglobulin M antibodies were present in both subclinical and symptomatic patients, five days after the onset of illness. Therefore, to enhance the sensitivity of detection, combination of the polymerase chain reaction and the immunoglobulin M enzyme-linked immunosorbent assay has been proposed.^13^

However, to facilitate the diagnostic process relating to COVID-19, 25 clinical trials have been registered in the ClinicalTrials.gov database as diagnostic tests, and these are shown in Table 2.^14^ These trials described diagnostic tests focusing on an immunoglobulin G antibody testing kit to detect the virus, lung ultrasound to diagnose the etiology of respiratory failure in a pediatric intensive care unit and nasopharyngeal swabs to identify associated risk factors. Apart from these tests, breath tests, blood tests, computed tomography scans, serological tests, ultrasonography, radiological detection, electrocardiogram and transthoracic echocardiography, cell phone-based auto-diagnosis systems, scanning chest X-rays and use of artificial intelligence algorithms on images, etc., were registered in clinical trials focusing on several diagnostic tests to detect the virus, determine patients’ health status and make risk assessments.

**Table 2.** Diagnostic tests for COVID-19 registered in clinical trials

Diagnostic test	Number of trials	Remarks
Breath test	1	This consists of noninvasive detection of pneumonia in the context of COVID-19 using gas chromatography.^14^
Data collection and rhinopharyngeal swab	1	Testing for SARS-CoV-2 and other respiratory pathogens by PCR via nasopharyngeal swabbing and IgM/IgG rapid serological tests.^14^
Electrocardiogram and transthoracic echocardiography	1	Systematic collection of cardiovascular data to study the incidence of myocarditis and coronaropathy events during COVID-19 infection.^14^
COVID-19 diagnostic test	1	This has the aim of comparing three tests: PCR, an antigenic rapid diagnostic orientation test (RODT) and a serological TROD.^14^
New QIAstat-Dx fully automatic multiple PCR detection platform	1	Automatic multiple PCR detection platform to test the enrolled patients.^14^ The reasonably designed experiments are used to verify the performance of the cartridge detection and prove its clinical application value.^14^
Scanning chest X-rays and performing AI algorithms on images	1	To identify the radiographs of patients with COVID-19 and those with influenza pneumonitis, with accuracy verified through COVID-19 tests.^14^
Nasopharyngeal swab	2	To assess the prevalence and incidence of COVID-19 infection in patients with chronic plaque psoriasis who are on immunosuppressive therapy.^14^
COPAN swabbing^*^ and blood sample collection	1	Used for immune protection and pathogenesis in SARS-CoV-2 and sampling can be delivered via existing research personnel from furloughed projects.^14^
Lung ultrasound	2	Used to diagnose the etiology of respiratory failure in a PICU.^14^
Thoracic CT scan	1	Used to evaluate the diagnostic performance of chest CT in screening for COVID-related lung injury.^14^
SARS-CoV-2 IgG antibody testing kit	2	An at-home fingerprick test for SARS-CoV-2 IgG antibodies, used for high-risk healthcare workers.^14^
Sampling salivary	1	Used to evaluate the performance of a detection test for diagnosing SARS-CoV-2.^14^
Titanium blood test	1	This is used to continue patient monitoring and identify those at greatest risk of implant-related issues in the absence of regular clinic visits.^14^
Electrocardiogram, telemetry, echocardiogram, laboratory values	1	These tests are done in order to identify cardiovascular manifestations of hospitalized patients with coronavirus disease 2019.^14^
Ultrasound lung imaging as part of FAST + evaluation	1	FAST adjunct evaluation in the trauma bay that can include lung parenchyma imaging at the initial assessment to help stratify patients into low or high-risk groups for active COVID-19 infection.^14^
Point-of-care ultrasonography (POCUS)	1	Used to analyze changes in the appearance of the lungs and heart through serial acquisition of focused point-of-care ultrasound images in a cohort of patients with or under investigation for COVID-19.^14^
Assessment of cardiovascular diseases and cardiovascular risk factors	1	Cardiovascular disease risk factors are defined as characteristics, both modifiable and non-modifiable, that increase the risk of developing CVD.^21^ SARS-CoV-2 infects host-cells via ACE2-receptors and leads to myocardial injury and chronic damage to the cardiovascular system.^14^
SAMBA II (Diagnostic for the Real World)	1	SAMBA II provides a simple and accurate system for diagnosing infection with SARS-CoV-2.
Cambridge Validated Viral Detection Method	1	This is a modified PCR test method for diagnosing infection within four hours, which is much faster than the current tests.
Biomarker expression	1	Used for clinical diagnosis among patients who develop a flu-like syndrome with fever and coughing.^14^
Standard screening strategy and new screening strategy	1	Designed to compare the screen accuracy and efficiency of two screening strategies.^14^
Odd/even birth year intervention groups	1	Used to measure the agreement between the detection of SARS-CoV-2 virus using a foam nasal swab tested directly after collection.^14^
Radiological detection	1	Used to detect chest X-ray and CT scan of viral infection in the lungs.^14^
Serology	1	Serology is the scientific study of serum and other body fluids. It is used for diagnostic identification of antibodies in the serum.
Recombinase-aided amplification (RAA) assay	1	Recombinase-aided amplification (RAA) assay is a novel isothermal nucleic acid amplification technique that can detect a variety of pathogens.^14^

PCR = polymerase chain reaction; IgM = immunoglobulin M; IgG = immunoglobulin G; AI = artificial intelligence; CT = computed tomography; FAST = focused assessment with sonography for trauma; CVD = cardiovascular disease; ACE2 = angiotensin-converting enzyme 2; VTM = viral transport media; PICU = pediatric intensive care unit.

^*^COPAN ITALIA SpA, Brescia, Italy.

One trial is using the Cambridge Validated Viral Detection Method, which is a modified polymerase chain reaction (PCR) test method that makes it possible to diagnose infection within four hours, which is much faster than the current tests. Another trial that has been registered is working to compare three tests that are currently available: PCR, antigenic rapid diagnostic orientation test and serological rapid tests for diagnostic orientation. Most of these registered trials will be finished by the end of 2020 and the successful trials will be able to facilitate the diagnostic process for COVID-19 patients.

### Therapeutics (drugs and treatment)

At present, COVID-19 pneumonia has no specific treatment. Therefore, the need for supportive care and preclusion of complications and nosocomial transmission has been emphasized by clinical managements. Oxygen should be provided as soon as possible to patients who experience respiratory distress. However, fluid replacement should be comparatively conservative unless there is any sign of hypoperfusion of tissue, since this can result in edema of the lungs and worsen the oxygen status. In addition, fluid replacement is an important concept within treatments for severe acute respiratory infections because of its ability to shorten the duration of ventilation. Systemic corticosteroids have the potential to delay clearance of viruses and so they are not generally recommended.

However, most of the drugs investigated in the present COVID-19 trials and treatments were basically designed for another bacterium. Several trials were started in order to test particular antibodies and vaccines, mainly targeting SARS-CoV-2. Here, these ongoing therapeutic options have been summarized.

Up to April 12, 2020, 188 clinical trials relating to for SARS-CoV-2 therapeutics had been registered in the clinical trials registry (ClinicalTrials.gov). These are reported in Table 3.^14^ Among these trials, 57 investigated antivirals, 57 antimalarials, 87 anti-inflammatories, 6 antiretrovirals, 13 dietary supplements, 21 standard treatment care, 9 traditional Chinese medicine, 6 oxygen and nitric acid therapy, 3 plasma, 11 antibodies, 26 antibiotics and several other therapeutics. Among these, some drugs, especially antiviral and antimalarial drugs, have shown effective results in ongoing treatment processes for COVID-19, and several patients have been successfully cured.^15^ On the other hand, in some cases, these drugs have also shown negative results.^16^ Thus, without proper results from successful clinical trials, specific therapeutics cannot be identified. However, most of these trials are expected to finish by the end of 2020, whereupon successful results from these trials will be able to assist in developing specific therapeutics for COVID-19 infection.

**Table 3.** Drugs for treating COVID-19 identified in registered clinical trials

Drugs	Number of trials	Remarks	Drug category
Thalidomide	2	Thalidomide has immune regulatory effects.^14^ Formula: C_13_H_10_N_2_O_4_	Anti-inflammatory
Naproxen	1	Used to treat pain and inflammatory diseases. Formula: C_14_H_14_O_3_	Anti-inflammatory
Ibuprofen	1	Used to treat pain, fever, and inflammation. Formula: C_13_H_18_O_2_	Anti-inflammatory
Escin	1	Used for treatment of chronic venous insufficiency. Formula: C_55_H_86_O_24_	Anti-inflammatory
Piclidenoson	1	Used for autoimmune-inflammatory disorders. Formula: C₁₈H₁₉IN₆O₄	Anti-inflammatory
Colchicine	4	Colchicine lessens the building up of uric acid crystals. Formula: C_22_H_25_NO_6_	Anti-inflammatory
CD24Fc	1	This is a biological immunomodulator. It addresses the major challenges associated with COVID-19.^14^	Anti-inflammatory
Aspirin	1	Used to reduce pain, fever, or inflammation. Formula: C₉H₈O₄	Anti-inflammatory
Hydrocortisone	1	Used as a replacement treatment for people whose adrenal glands are not producing enough natural cortisol.^14^ Formula: C_21_H_30_O_5_	Anti-inflammatory
ACE inhibitor	2	ACE inhibitors are used primarily for treatment of high blood pressure and heart failure.	Anti-inflammatory
Hyperbaric oxygen	1	Shows beneficial effects in various inflammatory diseases.^14^	Anti-inflammatory
Nitric oxide	5	This stimulates the release of certain hormones, such as insulin and human growth hormone.	Anti-inflammatory
N-acetylcysteine + Fuzheng Huayu tablet	1	N-acetylcysteine is a part of basic treatment. Fuzheng Huayu tablets have been proved effective in inhibiting MMP activity, to protect the subepithelial basement membrane.^14^	Anti-inflammatory
N-acetylcysteine + Placebo	1	N-acetylcysteine is a part of basic treatment. Placebo is used in clinical trials to test the effectiveness of treatments.	Anti-inflammatory
NORS (nitric oxide releasing solution)	1	NORS has the potential to decontaminate the upper respiratory tract.^14^	Anti-inflammatory
Lopinavir/ritonavir tablets combined with Xiyanping injection	1	Lopinavir/ritonavir is a promising candidate for both COVID-19 treatment and PEP.^14^ Xiyanping injection has anti-inflammatory and immune regulatory effects.	Anti-inflammatory
Darunavir	1	Used to treat and prevent HIV/AIDS. Formula: C_27_H_37_N_3_O_7_S	Antiretroviral
Immunoglobulin of cured patients	1	This acts as a critical part of the immune response by specifically recognizing and binding to antigens.^14^	Antiretroviral
Emtricitabine/tenofovir disoproxil	1	Used to treat HIV with a combination of two antiretroviral medications: tenofovir disoproxil and emtricitabine.	Antiretroviral
ASC09/ritonavir group	1	These are antiretroviral medications. A combination of them has been used for SARS-CoV-2 pneumonia.^14^	Antiretroviral
Ritonavir + oseltamivir	1	Ritonavir is used to treat HIV. This combination has been used for SARS-CoV-2 pneumonia.^14^	Antiretroviral
Lopinavir/ritonavir	15	Lopinavir has been used against HIV infections as a fixed-dose combination with another protease inhibitor, ritonavir. Formula: C_37_H_48_N_6_O_5_S_2_	Antiviral
Arbidol	2	Arbidol is pharmacodynamic in vitro against coronaviruses. Formula: C_22_H_25_BrN_2_O_3_S	Antiviral
Favipiravir	3	It has low toxicity (CC50 > 400 µM).^22^ Formula: C_5_H_4_FN_3_O_2_	Antiviral
Ribavirin	1	Used to treat hepatitis C and some viral hemorrhagic fevers. Formula: C_8_H_12_N_4_O_5_	Antiviral
Natural honey	1	Honey as a first-line treatment for acute cough caused by upper respiratory tract infection.^14^	Antiviral
Favipiravir combined with Tocilizumab	1	Favipiravir is used to treat influenza. Tocilizumab is used to treat rheumatoid arthritis.	Antiviral
Antiviral treatment and prophylaxis	1	The aim is to treat non-severe confirmed cases of COVID-19 and provide chemoprophylaxis for their contacts.^14^	Antiviral
Remdesivir	7	Nucleotide analog that inserts into viral RNA chains.	Antiviral
DAS181	5	This shows inhibitory activity against seasonal influenza.^23^	Antiviral
Placebo	39	A placebo is an inert substance or treatment that is designed to have no therapeutic value.	Other treatment/drug
Remdesivir placebo	2	Remdesivir is a nucleotide analog. Placebo is used with this in order to test the effectiveness of treatments.	Antiviral
Oseltamivir	4	This inhibits viral neuraminidase.^24^ Formula: C_16_H_28_N_2_O_4_	Antiviral
ASC09F + oseltamivir	1	Oseltamivir reduces the spread in the respiratory tract.^24^ ASC09F has been combined with oseltamivir to evaluate efficacy in relation to SARS-CoV-2 pneumonia.^14^	Antiviral
Combination of protease inhibitors, oseltamivir, favipiravir, and chloroquine	1	Used for antiviral treatment, orally. They are intended to have a systemic effect, reaching different parts of the body.	Antiviral
Arbidol hydrochloride	2	Used to prevent severe pneumonia and cytokine dysregulation induced by influenza viruses. Formula: C_22_H_26_BrClN_2_O_3_S	Antiviral and anti-inflammatory
Lopinavir/ritonavir + hydroxychloroquine	2	Lopinavir/ritonavir is a promising candidate for COVID-19 treatment.^14^ Hydroxychloroquine is used to prevent malaria in areas where malaria remains sensitive to chloroquine.	Antiviral and anti-inflammatory
Plaquenil	1	Used to prevent and treat malaria. Formula: C_18_H_26_ClN_3_O	Antimalarial
Chloroquine analog (GNS651)	1	This has been tested in patients with advanced or metastatic cancer who have SARS-CoV-2 infection that is not eligible for a resuscitation unit.^14^	Antimalarial and anti-inflammatory
Hydroxychloroquine + azithromycin	2	These are antiviral plus anti-inflammatory and are used for improving the efficacy of eradication of COVID-19 virus.^25^	Antiviral and anti-inflammatory
Hydroxychloroquine	38	Hydroxychloroquine is a drug that has been used to improve the clinical outcome from COVID-19.^25^ Formula: C_18_H_26_ClN_3_O	Antimalarial and anti-inflammatory
Chloroquine	4	Chloroquine is a medication primarily used to treat malaria in areas where malaria remains sensitive to its effects. Formula: C_18_H_26_ClN_3_	Antimalarial and anti-inflammatory
Chloroquine phosphate	4	Chloroquine phosphate is the phosphate salt of chloroquine with antimalarial and anti-inflammatory properties. Formula: C_18_H_32_ClN_3_O_8_P_2_	Antimalarial and anti-inflammatory
Hydroxychloroquine sulfate	9	Hydroxychloroquine sulfate works by reducing inflammation in people with autoimmune diseases.	Antimalarial and anti-inflammatory
Siltuximab	2	Siltuximab is a chimeric monoclonal antibody. Formula: C_6450_H_9932_N_1688_O_2016_S_50_	Antibody
Meplazumab for injection	1	This has the potential to mediate both treatment and prophylaxis of falciparum malaria.^14^	Antibody
Bevacizumab	2	This is an anti-VEGF recombinant monoclonal antibody. Formula: C_6638_H_10160_N_1720_O_2108_S_44_	Antibody
Nivolumab	1	This works as a checkpoint inhibitor.	Antibody
γ-globulin	1	A major class of immunoglobulins found in the blood.	Antibody
Mavrilimumab	1	Used to treat rheumatoid arthritis. Formula: C_6706_H_10438_N_1762_O_2104_S_54_	Antibody
Sarilumab (Kevzara) SC	1	Used by adult patients who are intolerant to biological or non-biological disease-modifying antirheumatic drugs.^26^	Antibody
Pembrolizumab (MK-3475)	1	Treatment of recurrent or metastatic cervical cancer. Formula: C_6534_H_10004_N_1716_O_2036_S_46_	Antibody
PD-1 blocking antibody + standard treatment	1	PD-1 acts as a negative regulator of T cell function. Monoclonal antibody blocking the activity of PD-1 can successfully reduce tumor load.^14^	Antibody
Azithromycin	16	Used for treatment of a number of bacterial infections. Formula: C_38_H_72_N_2_O_12_	Antibiotic
Carrimycin	1	This is effective against mycobacterium tuberculosis.^27^	Antibiotic
Ceftaroline	1	This is a cephalosporin antibiotic with anti-MRSA activity. Formula: C_22_H_21_N_8_O_8_PS_4_	Antibiotic
Macrolide	1	Used to inhibit bacterial protein synthesis.	Antibiotic
Ceftriaxone	1	Used to treat severe or life-threatening bacterial infections. Formula: C_18_H_18_N_8_O_7_S_3_	Antibiotic
Moxifloxacin	1	Used to treat bacterial infections of the lungs and stomach. Formula: C_21_H_24_FN_3_O_4_	Antibiotic
Levofloxacin	1	Used to treat bacterial infections, including pneumonia. Formula: C_18_H_20_FN_3_O_4_	Antibiotic
Amoxicillin-clavulanate	1	Used for treatment of a number of bacterial infections. Formula: C_24_H_28_N_4_O_10_S	Antibiotic
Atovaquone/azithromycin	1	Atovaquone is used to treat serious lung infection. Azithromycin is used to treat various bacterial infections.	Antibiotic
Piperacillin-tazobactam	1	The combination has activity against many Gram-positive and Gram-negative bacteria.	Antibiotic and inhibitor
Hydroxychloroquine sulfate + azithromycin	1	Hydroxychloroquine sulfate is an oral antimalarial medicine. Azithromycin is an antibiotic used for treatment of a number of bacterial infections.	Antimalarial and antibiotic
Vitamin C	6	Vitamin C acts as an antioxidant and helps to protect cells from damage caused by free radicals.	Dietary supplement
Vitamin D	3	Vitamin D allows the intestines to stimulate and absorb calcium and reclaim calcium.	Dietary supplement
Zinc	2	Zinc can significantly reduce risk of age-related infectious diseases and macular degeneration.	Dietary supplement
Glucose tablets	1	Used to treat hypoglycemia or low blood sugar.^14^ Formula: C_6_H_12_O_6_	Dietary supplement
Ascorbic acid	1	Used to treat low levels of vitamin C in people. Formula: C_6_H_8_O_6_	Dietary supplement
Interferon beta-1a	2	Interferon beta-1a is used to treat multiple sclerosis. Formula: C_908_H_1408_N_246_O_252_S_7_	Interferon
Interferon beta-1b	1	Used to treat relapsing/remitting multiple sclerosis. Formula: C_908_H_1408_N_246_O_253_S_6_	Interferon
Recombinant human interferon α1β	2	Applied in the initial treatment and prevention of SARS and MERS.^14^	Interferon
Alpha-interferon nebulization	1	This mobilizes the body’s immune system to fight cancer.	Interferon
Peginterferon lambda-1a	1	Pegylated type III interferon with marked anti-HCV activity which is mainly used for treatment of CHB.^28^	Interferon
Arbidol hydrochloride combined with interferon atomization	1	They are combined to treat SARS-CoV-2 viral pneumonia, so as to provide reliable evidence-based medicine for treating viral pneumonia.^14^	Antiviral and interferon
Huaier granules	1	Orally bioavailable traditional Chinese medicine (TCM) composed of granules containing an aqueous extract of *Trametes robiniophila* Murr (Huaier).	Traditional Chinese medicine
T89	1	T89 is a botanical drug for oral use. T89 can provide substantial benefits in the prevention or alleviation of symptoms associated with acute mountain sickness.	Traditional Chinese medicine
TCM	2	Traditional medicine includes various forms of herbal medicine, acupuncture, cupping therapy, gua sha, massage, bonesetter, exercise and dietary therapy.	Traditional Chinese medicine
Yinhu Qingwen granula	2	Yinhu Qingwen granula consists of 11 common nontoxic traditional Chinese medicines and previous vivo antiviral studies showed its activity for inhibition of COVID-19.^14^	Traditional Chinese medicine and antiviral
Xiyanping injection	1	Xiyanping is a TCM preparation with andrographolide as a principal component; it has significant antibacterial and antiviral effects.^29^	Traditional Chinese medicine and antiviral
YinHu QingWen decoction	2	This consists of 11 common nontoxic traditional Chinese medicines such as *Polygonum cuspidatum*, Honeysuckle, Nepeta, *Ligustrum lucidum*.^14^	Traditional Chinese medicine and antiviral
PUL-042 inhalation solution	2	This reduces the infection rate and progression to COVID-19 in adults exposed to SARS-COV-2.^14^	Inhaler
Ciclesonide metered dose inhaler	1	It is used to treat asthma and allergic rhinitis. Formula: C_32_H_44_O_7_	Inhaler
Levamisole pill + budesonide + formoterol inhaler	1	Levamisole can increase lymphocytes.^14^ Budesonide can suppress the immune reaction locally in the respiratory system.^14^ Formoterol is a β2 agonist and can open airways.^14^	Inhaler
Sarilumab	6	Used for the treatment of rheumatoid arthritis. Formula: C_6388_H_9918_N_1718_O_1998_S_44_	IL-6 receptor blocker
RoActemra IV	1	This is a first-in-class anti-IL-6 receptor (aIL-6R) therapy. IL-6 plays a key role in activating the inflammatory pathway.	IL-6 receptor blocker
Losartan	5	Losartan is an oral medication mainly used to treat high blood pressure. It may be used alone or in addition to other blood pressure medications.	Angiotensin receptor blockers
Valsartan	1	Valsartan is an oral medication used to treat high blood pressure, heart failure and diabetic kidney disease.^14^ Formula: C_24_H_29_N_5_O_3_	Angiotensin II receptor blocker
Anakinra	3	Used to treat rheumatoid arthritis. Formula: C_759_H_1186_N_208_O_232_S_10_	Receptor antagonist
Sargramostim	1	Sargramostim is a recombinant granulocyte macrophage colony-stimulating factor (GM CSF). Formula: C_639_H_1006_N_168_O_196_S_8_	Biological response modifier
Methylprednisolone	5	Used to suppress the immune system. Formula: C_22_H_30_O_5_	Corticosteroid
Dexamethasone	2	Used to reduce the duration of mechanical ventilation.^14^ Formula: C_22_H_29_FO_5_	Corticosteroid
Fingolimod	1	This is an effective immunological modulator. Formula: C_19_H_33_NO_2_	Immunomodulator
Clopidogrel	1	Used to reduce the risk of heart disease. Formula: C_16_H_16_ClNO_2_S	Inhibitor
Rivaroxaban	1	Used to treat and prevent blood clots. Formula: C_19_H_18_ClN_3_O_5_S	Inhibitor
Baricitinib	3	Used for the treatment of rheumatoid arthritis in adults. Formula: C_16_H_17_N_7_O_2_S	Inhibitor
Sildenafil citrate tablets	1	This is an oral therapy for erectile dysfunction. Formula: C_22_H_30_N_6_O_4_S	Inhibitor
Atorvastatin	1	Used to prevent cardiovascular disease. Formula: C_33_H_35_FN_2_O_5_	Inhibitor
Omeprazole	1	Used in treating gastroesophageal reflux disease. Formula: C_17_H_19_N_3_O_3_S	Inhibitor
Ruxolitinib	3	Used to treat myelofibrosis and polycythemia vera. Formula: C_17_H_18_N_6_	Inhibitor
Cobicistat	1	Used to treat human immunodeficiency virus infection. Formula: C_40_H_53_N_7_O_5_S_2_	Inhibitor
Camostat mesylate	2	Camostat mesylate can block entry of SARS-CoV-2 into cells. Formula: C_20_H_22_N_4_O_5_	Inhibitor
Tofacitinib	1	Tofacitinib can mitigate alveolar inflammation.^14^ Formula: C_16_H_20_N_6_O	Inhibitor
ARB/ACEI	1	Used to treat high blood pressure.	Inhibitor
Angiotensin 1-7	1	Angiotensin 1-7 is a vasodilator agent. Formula: C_41_H_62_N_12_O_11_	Inhibitor
RhACE2 APN01	1	This is believed to have the potential to inhibit COVID-19 infection and reduce lung injury.	Inhibitor
Nintedanib 150 mg	1	Used for treatment of idiopathic pulmonary fibrosis. Formula: C_31_H_33_N_5_O_4_	Inhibitor
Calcium channel blockers	1	Used to relax blood vessels and increase the supply of blood and oxygen to the heart.	Antihypertensive
Tranexamic acid	2	Used to treat excessive blood loss from major trauma. Formula: C_8_H_15_NO_2_	Antifibrinolytic
Plasma	2	This helps to distribute heat throughout the body.	Plasma
Hyperimmune plasma	1	This can induce high serum concentrations of antibodies against Gram-negative LPS.^30^	Plasma
Tocilizumab	13	Used for treatment of rheumatoid arthritis. Formula: C_6428_H_9976_N_1720_O_2018_S_42_	Other treatment/drug
BLD-2660	1	Used to reduce viral replication.^14^	Antifibrotic
Telemedicine	1	This allows healthcare professionals to evaluate, diagnose and treat patients using telecommunications technology.	Other treatment/drug
Oxyhydrogen	1	This is an adjuvant therapy for patients infected with COVID-19 pneumonia, for improving the clinical symptoms.^14^	Other treatment/drug
Best supportive care (BSC) + IFX-1	1	This is a phase II study with two treatment arms. It is used in patients with severe COVID-19 pneumonia.^14^	Other treatment/drug
Usual practice + Symbicort Rapihaler	1	Interventional patient will be treated with Symbicort Rapihaler, which is inhaled into the lungs to treat asthma.^14^	Other treatment/drug
Thymosin + standard treatment	1	Thymosin is used to regulate cellular immunity in sepsis patients.^14^	Other treatment/drug
Oxygen treatment	1	Oxygen treatment delivers oxygen gas for breathing.	Other treatment/drug
Physiological saline solution	1	A sterile solution of sodium chloride that is isotonic to body fluids; used to maintain living tissue temporarily.	Other treatment/drug
Aviptadil via intravenous infusion + maximal intensive care	1	Aviptadil is an analog of vasoactive intestinal polypeptide for treating erectile dysfunction. Maximal intensive care has been used for COVID-19-induced acute respiratory distress syndrome.^14^	Other treatment/drug
Normal saline infusion + Maximal intensive care	1	Maximal intensive care is defined not to include extracorporeal mechanical oxygenation.^14^ This combination has been tested in relation to coronavirus infection.^14^	Other treatment/drug
Discontinuation and continuation of RAS blocker therapy	1	It is crucial to determine whether RAS blockers should be discontinued or not in patients with COVID-19.^14^	Other treatment/drug
Thiazide or thiazide-like diuretics	1	These are widely used for management of hypertension.	Other treatment/drug
Angiotensin receptor blocker	1	Angiotensin receptor blockers are medications that block the action of angiotensin II and allow arteries and veins to widen.	Other treatment/drug
Thymosin alpha 1	1	This is a peptide fragment derived from prothymosin alpha, a protein that in humans is encoded by the PTMA gene.	Other treatment/drug
Bromhexine 8 mg	1	Used to treat chest congestion and coughing. Formula: C_14_H_20_Br_2_N_2_	Other treatment/drug
Anluohuaxian	1	Used to block the progression of pulmonary fibrosis and improve lung function in patients with COVID-19.^14^	Other treatment/drug
Eicosapentaenoic acid gastro-resistant capsules	1	This is a hydrolytic breakdown product of eicosapentaenoyl ethanolamide. Formula: C_20_H_30_O_2_	Other treatment/drug
Tradipitant	1	Used to treat motion sickness and atopic dermatitis. Formula: C_28_H_16_ClF_6_N_5_O	Other treatment/drug
RoActemra SC	1	This is an anti-human monoclonal antibody of the immunoglobulin G1 (IgG1) subclass.	Other treatment/drug
Defibrotide injection	1	This works by preventing formation of blood clots.	Other treatment/drug
Sterile water for injection	1	This preparation is designed solely for parenteral use, after addition of drugs that require dilution.	Other treatment/drug
Standard treatment/ medical care/ therapy	21	Standard therapy is the medical treatment that is normally provided to people with a given condition.	Other treatment/drug
Intravenous immunoglobulin	1	Intravenous immunoglobulin (IVIG) therapy can improve the prognosis for critically ill patients with SARS-CoV-2.^14^	Other treatment/drug
Oxygen therapy	1	Oxygen therapy is a treatment that provides supplemental oxygen.	Other treatment/drug
Deferoxamine	2	Used to treat transfusion-related chronic iron overload. Formula: C_25_H_48_N_6_O_8_	Iron chelator

ACE = angiotensin-converting enzyme; MMP = matrix metallopeptidases; PEP = post-exposure prophylaxis; RSV = respiratory syncytial virus; RNA = ribonucleic acid; VEGF = vascular endothelial growth factor; GM-CSF = granulocyte-macrophage colony-stimulating factor; PD-L1 = programmed death-ligand 1; PD-1 = programmed cell death protein 1; MRSA = methicillin-resistant *Staphylococcus aureus;* HCV = hepatitis C virus; CHB = complete heart block; IL-6 = interleukin 6; RA = rheumatoid arthritis; ARDS = acute respiratory distress syndrome; CYP3A = cytochrome P450 3A; JAK1/3 = Janus kinase 1/3; ARB = angiotensin receptor blocker; ACEI = angiotensin-converting enzyme inhibitors; RAS = renin-angiotensin system; LPS = lipopolysaccharides; PTMA = prothymosin alpha; SC = subcutaneously; IV = intravenously; CC50 = cytotoxic concentration 50%.

### Vaccine and biological trials

With the rise of SARS-CoV-2, around 30 potential ongoing trials on vaccines have been classified in the registers of ClinicalTrials.gov (Table 4). A variety of technologies, including use of deoxyribonucleic acid (DNA)-based techniques, messenger ribonucleic acid (RNA)-based techniques, synthetic particles, nanoparticles and modified virus-like particles have been used. It will most probably take around a year for phase 1 clinical trials to begin in relation to a large proportion of the candidate vaccines, unless funded by the Coalition for Epidemic Preparedness Innovations (CEPI). However, a kit that was developed by Beijing Genomics Institute (BGI) passed the emergency approval process of the National Medical Products Administration of China and so it is currently being used in clinical and surveillance centers in China.^17^ All of these trials are testing the immunogenicity and safety of their corresponding vaccine candidates relating to MERS-CoV, but have been excluded because of the unavailability of results so far. These trials are projected to be finished by December 2020 (two studies in Russia) and by December 2021 (in Germany).^18^^,^^19^

**Table 4.** Biologics and vaccines for use against COVID-19 identified in registered clinical trials

Biologics/vaccines	Number of trials	Remarks
NK cells	2	NK cells are essential for innate immunity and adaptive immunity.^14^
IL-15-NK cells	1	These show improved pharmacokinetic characteristics.^14^
NKG2D CAR-NK cells	1	NK cells modified by CAR have been demonstrated to be very safe without severe adverse events such as cytokine-releasing syndromes.^14^
ACE2 CAR-NK cells	1	These inhibit SARS-CoV-2 infection in type II alveolar epithelial cells.^14^
NKG2D-ACE2 CAR-NK cells	1	NKG2D-ACE2 CAR-NK cells are derived from cord blood and are used for providing safe and effective cell therapy for COVID-19.^14^
NestCell	1	NestCell is a mesenchymal stem cell therapy produced by Cellavita.^14^
WJ-MSCs	1	WJ-MSCs have been derived from cord tissue of newborns; screened for HIV1/2, HBV, HCV and CMV; and cultured to enrich for MSCs.^14^
MSCs	2	MSCs can significantly reduce pathological changes in lungs.^14^
Saline containing 1% human serum albumin (solution of MSC)	1	Human serum albumin is the serum albumin found in human blood and it is the most abundant protein in human blood plasma. Saline containing 1% human serum albumin has been tested for use against severe COVID-19.^14^
Pathogen-specific aAPC	1	aAPCs modified with lentiviral vector expresses synthetic minigenes based on domains of selected viral proteins.^31^
LV-SMENP-DC vaccine	1	LV-SMENP-DC vaccine is made by modifying DC with lentivirus vectors expressing COVID-19 minigene SMENP and immune modulatory genes.^14^
Antigen-specific CTLs	1	Used to produce autologous cell products for adoptive cell therapy.
Recombinant novel coronavirus vaccine (adenovirus type 5 vector)	1	This is currently being investigated for prophylaxis against SARS-CoV-2.
Dental pulp mesenchymal stem cells	1	These are tissue-specific adult stem cells and can undergo directed differentiation to multiple cell lineages including odontoblasts, osteoblasts, chondrocytes and adipocytes.
BCG vaccine	2	BCG vaccine is a vaccine primarily used against tuberculosis. It has broad power to boost the immune system against the novel coronavirus.
CAStem	1	CAStem is an injectable product composed of immunity and matrix-regulatory cells (IMRCs).^14^
Emapalumab	1	This is an anti-interferon-gamma antibody used for treatment of hemophagocytic lymphohistiocytosis, which currently has no cure.
Anakinra	1	Anakinra is a biopharmaceutical drug used as a second-line treatment to manage symptoms of rheumatoid arthritis after treatment with a disease-modifying antirheumatic drug (DMARD) has failed.
Human amniotic fluid	1	This is used for administration of amniotic fluid in SARS-CoV-2-positive patients.^14^
ChAdOx1 nCoV-19	1	ChAdOx1 nCoV-19 is a vaccine currently being investigated for prophylaxis against SARS-CoV-2.
Anti- SARS-CoV-2 plasma	1	This is collected through pheresis from volunteers who have recovered from COVID-19 disease.^14^
SARS-CoV-2 nonimmune plasma	1	This is the standard plasma collected prior to December 2019 to evaluate the efficacy of treatment among adults exposed to COVID-19.^14^
UC-MSCs	3	UC-MSCs are a class of cells with significant self-renewal and multi-lineage differentiation properties.^14^
Allogeneic human dental pulp stem cells (BSH BTC and Utooth BTC)	1	Used for routine treatment and intravenous injection.^14^ The safety and efficacy of these cells have been evaluated in relation to treatment of severe pneumonia caused by COVID-19.^14^
Umbilical cord Wharton’s jelly derived human MSCs	1	If all MSCs share several characteristics regardless of the tissue source, the highest productions of bioactive molecules and the strongest immunomodulatory properties are yielded by those from Wharton’s jelly of the umbilical cord.^14^
Blood sampling	1	This is used to determine ACE2 levels and activity in patients with SARS-CoV-2 infection who are admitted to an intensive care unit.^14^
High-titer anti-SARS-CoV-2 plasma	1	This is an option for COVID-19 treatment and may be available from people who have recovered and can donate plasma.^14^
mRNA-1273	1	mRNA-1273 is a novel lipid nanoparticle (LNP)-encapsulated mRNA-based vaccine.^14^
SARS-CoV-2 PCR	1	This is used to evaluate the virological and clinical outcomes among subjects exposed to contacts presenting high or moderate risk of SARS-CoV-2 transmission.^14^
bacTRL-spike	1	This has been engineered to deliver plasmids containing synthetic DNA-encoding spike proteins from SARS-CoV-2.^14^
MSC-derived exosomes	1	These enable significantly reduced lung inflammation and pathological impairment resulting from different types of lung injury.^14^
Anti-SARS-CoV-2 convalescent plasma	2	This is an option for treatment of COVID-19 and may be rapidly available when there are sufficient numbers of people who have recovered and can donate high-titer neutralizing immunoglobulin-containing plasma.^14^

NK = natural killer; IL-15 = interleukin-15; ACE2 = angiotensin-converting enzyme 2; WJ-MSCs = mesenchymal stem cells from Wharton’s jelly; MSCs = mesenchymal stem cells; HIV = human immunodeficiency virus; HBV = hepatitis B virus; HCV = hepatitis C virus; CMV = cytomegalovirus; CTLs = cytotoxic T lymphocytes; BCG = bacille Calmette-Guérin; ChAdOx1 = chimpanzee adenovirus Oxford 1; UC-MSCs = umbilical cord-derived mesenchymal stem cells; mRNA = messenger ribonucleic acid.

At present, vaccines for SARS-CoV-2 are still at the development stage and none are at the testing stage. On January 23, 2020, an announcement was made by the Coalition for Epidemic Preparedness Innovations (CEPI) that vaccine development programs will be funded by them in partnership with Moderna, University of Queensland and Inovio, with the aim of clinically testing the experimental vaccines within 16 weeks. The vaccine candidates will be developed using the DNA, recombinant and mRNA vaccine platforms of these organizations.^20^

Among the trials identified, vaccines based on the following are expected to show high potential as effective vaccines against COVID-19: natural killer (NK) cell group; mesenchymal stromal cell (MSC) group; bacille Calmette-Guérin (BCG); LV-SMENP-DC; CAStem; chimpanzee adenovirus Oxford 1 (ChAdOx1); aAPC; mRNA-1273; bacTRL-Spike; etc. However, convalescent plasma, high-titer anti-SARS-CoV-2 plasma, SARS-CoV-2 non-immune plasma and high-titer anti-SARS-CoV-2 plasma are bio-pharmaceutical products that have also been identified in clinical trial as vaccine candidates and are expected to have high potentiality to act against COVID-19. It would then be possible to successfully apply these vaccines if positive results are obtained from these registered trials.

### Devices and products

To facilitate the treatment process relating to COVID-19 infection, several trials to develop device and products have been registered. In total, 31 trials relating to devices and products for COVID-19 had been registered up to April 12, 2020, which is more than the numbers of diagnostic test trials and vaccine trials. Most of the devices in these trials related to oxygen supply and monitoring, sensors, image processing, high-flow nasal cannulas, inspiratory and expiratory training devices, MAGEC spine rods (NuVasive, California, United States), echocardiography devices, transcatheter aortic valve replacement (TAVR) or surgical aortic valve replacement (SAVR) and apps for COVID-19 patients. All these devices are expected to be highly effective for treating COVID-19 patients. Therefore, before applying these devices rapidly, the medical world needs to wait until positive finished results are received, in order to avoid any kind of negative effects on patients (Table 5).

**Table 5.** Devices and products for use against COVID-19 identified in registered clinical trials

Devices/products	Number of trials	Remarks
Web application	1	Used to assess the evolution of the number of calls to the emergency service within 12 days after the launch of the application.^14^
Hyperbaric oxygen therapy (HBOT) device	1	This delivers pure oxygen in a pressurized room or tube.^14^
GO2 PEEP mouthpiece	2	This effectively delivers PEEP with every breath of patients.^14^
Oxyhydrogen device	1	Used to improve symptoms of patients with COVID-19.^14^
Oxygen monitoring device	1	Used for monitoring peripheral blood oxygen saturation and oxygen concentration.^14^
CT-V	1	Used to detect parenchymal lung function changes at a voxel level.^14^
vv-ECMO + cytokine adsorption (CytoSorb adsorber)	1	This combination can lead to reduction of the levels of circulating pro and anti-inflammatory cytokines.^14^
vv-ECMO only (no cytokine adsorption)	1	This provides support for the lungs. It is used in situations of acute respiratory failure in COVID-19-disease.^14^
VivaDiag^*^ COVID-19 lgM/IgG	1	This is used to evaluate the immune response of negative patients during the outbreak of COVID-19.^14^
A mindfulness meditation mobile app - Calm v4.22	1	This app includes meditation lessons, sleep stories (bedtime stories for grown-ups), sleep music and nature sounds.^14^
Medical/surgical mask	2	This is a loose-fitting disposable device for creating a physical barrier.
N95 respirator	1	This is a respiratory protective device.
High-flow nasal cannula (HFNC)	1	This is a device that can deliver 100% humidified and heated oxygen.
Inspiratory training device	1	This is a device that is used to determine the effectiveness and safety of respiratory training in relation to preventing and reducing the severity of COVID-19.^14^
Expiratory training	1	This tends to improve coughing and reduce the sensation of respiratory effort.^32^
Cordio app v1.54	1	This app uploads vocal data to the sponsor’s servers for analysis.^14^
Biosensors	1	These detect changes in respiration, temperature and circulation.^14^
SensiumVitals^**^ wearable sensor	1	This sensor measures heart rate, respiratory rate, temperature and suspected coronavirus in a designated location (e.g. a hotel).^14^
CVVH machine	1	This has the aim of clearing CO_2_ and improve oxygenation.^14^
MAGEC spine rod^***^	1	Magnetically controlled spine rod for treatment of scoliosis.^14^
Transpulmonary thermodilution	1	Transpulmonary thermodilution is a technique that provides a full hemodynamic assessment.^33^
Echocardiography	1	Echocardiography is a test that uses sound waves to produce live images of the heart.
TAVR or SAVR	1	Used to describe rates of morbidity and mortality.^14^
CPAP treatment device	1	CPAP is a common treatment for obstructive sleep apnea.
Home blood pressure monitoring device - Qardio Arm (Qardio, California, United States)	1	Home blood pressure device with telemonitoring capability that allow participants and their physicians to monitor blood pressure over time and to titrate blood pressure medications as needed for persistently elevated blood pressure.^14^
Automated oxygen administration - FreeO2 device (OxyNov Inc, Quebec, Canada)	1	This provides a solution for reducing the number of interventions by healthcare workers relating to oxygen therapy, so as to reduce complications relating to oxygen and improve monitoring.^14^
CELLECTRAÂ^****^ 2000	1	This is applied to increase the permeability of cell membranes to enhance the uptake of drugs or vaccines into target cells.^34^
Caption AI	1	Software program to take the best possible pictures of the heart.^14^
Stem Cell Educator-Treated Mononuclear Cell Apheresis (Tianhe Stem Cell Biotechnologies Inc, Shandong, China)	1	This circulates a patient’s blood through a blood cell separator, briefly cocultures the patient’s immune cells with adherent CB-SC in vitro, and returns the “educated” autologous immune cells to the patient’s circulation.^14^
COVID-19 Symptom Tracker app v0.3	1	This records and monitors the symptoms of COVID-19 coronavirus infection; tracking in real time how the disease progresses.^14^

PEEP = positive end-expiratory pressure; CT = computed tomography; vv-ECMO = veno-venous extracorporeal membrane oxygenation; IgM = immunoglobulin M; IgG = immunoglobulin G; COPD = chronic obstructive pulmonary disease; CVVH = continuous veno-venous hemofiltration; NCP = novel coronavirus pneumonia; TAVR = transcatheter aortic valve replacement; SAVR = surgical aortic valve replacement; AI = artificial intelligence; CB-SC = cord blood stem cells.

^*^Everest Links Pte Ltd, Midview City, Singapore; ^**^Sensium Healthcare Ltd, Oxford, United Kingdom; ^***^NuVasive, California, United States; ^****^Inovio Pharmaceuticals, Pennsylvania, United States.

### Behavioral and other clinical trials

In order to facilitate the treatment process for COVID-19 infection, 65 behavioral and other trials were registered up to April 12, 2020. Most of these behavioral and other trials relate to guidelines, management, healthcare, surveys on anxiety, mood and quality of life and human biological samples, which are very necessary in relation to COVID-19 patients. Standard public health measures have been used to isolate patients and do contact tracing as per national guidelines.^14^ Video-based aerobic exercises have been used to increase physical activity levels, psychological condition and physical wellbeing.^14^ Blood sampling is necessary in order to detect COVID-19 seroconversion among medical and paramedical staff. Retrospective analysis is used in order to clearly understand the impact factors of clinical outcomes among hospitalized patients.^14^ Pulmonary ultrasound is used to assess the risk of severe clinical outcomes in patients with suspected or diagnosed COVID-19.^14^ The SPIN-CHAT software is used to evaluate videoconference-based interventions that are designed to improve the symptoms of anxiety and other mental health outcomes.^14^ Moreover, all the trials are expected to be highly effective if the results are positive with regard to treating COVID-19 patients. Additionally, before applying the results in practice, further experiments and studies should be done, to avoid any harmful effects or adverse events in relation to patients.

## DISCUSSION

This study was based on the database of ClinicalTrials.gov up to April 12, 2020. Most of the trials are being conducted in the United States and China. Since COVID-19 has spread all over the world, there is a growing need to also conduct investigations in other countries that have been affected. Moreover, most of the trials are in phase 2 and some trials have longer expected completion times.

SARS-CoV-2 is a very dangerous virus that is rapidly spreading all over the world. For effective solutions to be obtained quickly, trials should be completed within a short time. However, regulatory authorities need to carefully maintain proper recruitment protocols for clinical trials.

Observational studies account for slightly more than one-fourth of the total number of studies. This proportion needs to be increased somewhat, because observational studies directly focus on treatment protocols for COVID-19 patients. More studies should be conducted with numbers of participants above 1000, in order to find more accurate results. Since every person is important for proper investigation, more people with ages below 18 should be included.

The largest proportion of the trials relates to drugs. However, there need to be greater numbers of trials relating to other categories. Although antiviral drugs (remdesivir and lopinavir/ritonavir) and antimalarial drugs (especially hydroxychloroquine), plasma therapy, anti-inflammatory drugs and azithromycin have been investigated in the highest proportion of the trials, no accurate results that can be completed early have yet been found with regard to combating COVID-19. Moreover, some of the drugs investigated may have serious adverse events. Therefore, adequate precautions should be taken before applying a drug, to avoid any negative impacts. Successful conclusions from these trials are important and the results are expected to be received mostly at the end of 2020.

Since COVID-19 has a very high transmission rate, diagnostic tests are very important. Through these tests, people with the virus can be isolated. Otherwise, the virus may spread very quickly.

Polymerase chain reaction (PCR) tests, immunoglobulin G antibody testing kits and serological tests have been registered for trials in high numbers. However, more clinical trials are still needed in order to identify more efficient testing processes that have low cost and high detection rates within a short time, to control the transmission rate. Vaccines can be very effective to protect people from COVID-19, so more importance should be given to finding at least one effective vaccine as soon as possible. Overall, use of convalescent plasma, high-titer anti-SARS-CoV-2 plasma and SARS-CoV-2 non-immune plasma may show potential in relation to vaccines for treating COVID-19.

Moreover, to facilitate the treatment process, more effective devices, especially for oxygen therapy and patient monitoring systems, are important. Additionally, behavioral and other trials are also needed in order to understand and analyze healthcare management for COVID-19 and its impact on society, patients and medical science.

The world is now counting the days, in the hope of receiving positive successful results from the ongoing clinical trials as soon as possible, to combat COVID-19.

## CONCLUSIONS

This review found 339 clinical trials that evaluated interventions for preventing or treating coronavirus. Overall, use of antiviral drugs (remdesivir and lopinavir/ritonavir) and antimalarial drugs (especially hydroxychloroquine), plasma therapy, anti-inflammatory drugs and azithromycin may present some benefits for treating COVID-19 infection. Polymerase chain reaction (PCR) tests, immunoglobulin G antibody testing kits and serological tests are the diagnostic tests that are involved in the highest numbers of trials registered for detecting COVID-19. Moreover, several kinds of plasma and bio-pharmaceutical products identified in trials may present potential as candidate vaccines against COVID-19. Additionally, trials on devices (oxyhydrogen devices, patient monitoring devices, etc.) and other clinical trials (surveys, behavioral trials and observational trials) may also have potential to facilitate the treatment process for COVID-19. However, completion results from the trials described in the present study are needed before any diagnostic test, therapeutics, vaccines, devices or other objects relating to clinical management processes for COVID-19 can be properly recommended. More randomized controlled trials are still necessary, in order to reduce the uncertainties regarding most clinical questions that surround COVID-19.

## LIMITATIONS

This study had some limitations. It was conducted within time limits and details of some trials were not properly available.
